# Contact Effects in thin 3D-Topological Insulators: How does the current flow?

**DOI:** 10.1038/srep09479

**Published:** 2015-03-30

**Authors:** Gaurav Gupta, Mansoor Bin Abdul Jalil, Gengchiau Liang

**Affiliations:** 1 Department of Electrical and Computer Engineering, National University of Singapore, Singapore 117576

## Abstract

The effect of different contact configurations (semi-infinite extended-channel, normal metal and ferromagnetic metal) on quantum transport through thin Bi_2_Se_3_ three-dimensional (3D) topological insulator (TI) slab (channel) has been investigated through Non-Equilibrium Green Function. The issue of contact dependent current flow and distribution across quintuple layers of 3D-TI has been addressed in this work and applied to expound the explanation for recent experimental work on electrical detection of spin-momentum locking on topological surface for long channel device. A theoretical model is propounded to develop a microscopic understanding of transport in 3D-TI in which contact type and magnetization concur with helical surface states of the TI channel to manifest seemingly counter-intuitive current distribution across layers. The quantum transport calculations for short channel devices with magnetic source and drain contacts postulate negative surface current for anti-phase magnetization whose axis is transverse to both current and quintuple layers. For in-phase magnetization at the two terminals, it is shown that observations can change fundamentally to result in anomalous current distribution. Such results are explained to stem from the confinement of 3D-TI between ferromagnetic contacts along the transport direction. A simple mechanism to validate topological insulators via quantum transport experiments has also been suggested.

The peculiar suppression of backscattering^1^ of helical fermions in odd-number^2^ of gapless Dirac^3^ surface bands of three-dimensional (3D) topological insulators (TI)^2^,^4^, accompanied with an insulating bulk, has recently drawn tremendous interest for Very-Large Scale Integration (VLSI) interconnect^5^, spintronic^6^,^7^ and quantum computing^8^ applications. Time-reversal symmetry (TRS) renders this protection to surface states against scattering from non-magnetic impurities and vacancies because in these states the spin of an electron is locked to its momentum vector, which results in a distinct momentum-space spin-texture, and therefore without spin-flip mechanism (e.g. doping with magnetic impurities) or breaking of TRS (e.g. application of a perpendicular magnetic field) an electron cannot be backscattered. The spin-texture which is left (right) handed for top-surface (bottom-surface) conduction band (see Fig. 1(a)) and vice-versa for valence bands has recently been shown to engender spin-polarized surface current^9^,^10^ with an average polarization transverse^11^ to non-equilibrium transport direction. Kramer's degeneracy theorem^12^,^13^ posits that these surface bands with antagonistic spin-texture on opposite surfaces are degenerate. If 3D-TI is thin enough, but thicker than 5 quintuple layers^9^ (QL) (1 QL ~ 0.943 nm) to eschew crossover to two-dimensional limit^14^, the wavefunction overlap between opposite surfaces then enables an electron to be backscattered from forward moving state of one surface to backward moving state of opposite one. However, for very large thickness a sizeable fraction of current flows through the bulk layers which weakens the signature of topological surface in electrical transport^9^. Thin 3D-TI (thickness ~ 10 nm^15^) with weak inter-surface coupling but surface dominant transport is therefore especially interesting and important^16^. We show in this work that this thickness regime may also provide a simple way to validate spin-momentum locking on topological surfaces through quantum transport experiments, in comparison to more complicated Berry-phase matching^17^ and optical (Spin-ARPES^18^ and Circular Dichroism^19^) experiments.

Furthermore, for the observation of the topological properties the device should be electronically operated (i) at small channel bias with Fermi-level (*E_F_*) close to the Dirac-point, to avoid the dominance of trivial bulk bands in observed results; and (ii) at low temperature to circumvent phonon scattering^20^. Physically, defect-free or compensated^21^ 3D-TI of short channel must be used because strong spin-charge coupling^22^,^23^ in 3D-TI would limit the spin-relaxation length^24^ to the mean-free path. Small channel length becomes especially important for the study of novel magneto-electric effects that may arise in 3D-TI due to ferromagnetic contacts^23^,^25^. Most experiments have, howbeit, been performed on very long channel devices^9^,^26^ because placing multiple probes for measurements that can accurately characterize the carrier transport become very challenging for small channel lengths. Therefore, despite of recent emphasis on the importance of short channel for investigating topological insulators, study of quantum transport and current distribution through such a device is completely lacking. For short-channel device the issue is further exacerbated by the confinement along transport direction. We show in this work that confinement especially due to ferromagnetic contacts can radically influence the experimental investigation of transport in 3D-TI. In this work, therefore, we carry out a systematic and comprehensive study of quantum transport and current distribution in thin 3D-TI based on Bi_2_Se_3_ for different contact configurations, which not only enables us to explain recent experimental work on electrical detection of spin-momentum locking on topological surface for long channel device, but also allows us to make new predictions for short-channel TI device like negative surface current and an exotic current distribution which may not be expected from conventional understanding of spin-texture for surface states of 3D-TI. Therefore, in this work, first a simple system with ‘Ferromagnetic Metal source-TI Channel-Extended TI Drain’ (FM-TI-exTI) is comprehensively examined to understand the recently published experimental data^9^ (long channel device). We then present a more microscopic analysis for different combinations of three different contact-types i.e. ferromagnetic metal (FM), normal metal (NM) and extended-TI (exTI), which finally evolves into more complicated results predicted in this work for short-channel devices with both metallic source and drain contacts where quantum effects may manifest exotic results and provide entirely new ways of identifying a topological insulator via electrical transport experiments.

The paper is organized as follows. Subsequent to this introduction, methods section describes the Hamiltonian and NEGF formalism implemented in this work for appraising different contacts. Results and Discussion section then presents our results on the effect of contacts on transport and more specifically on current distribution in 3D-TI. Finally, we conclude our findings with suggestions for experiments.

## Methods

**Figure 1:**
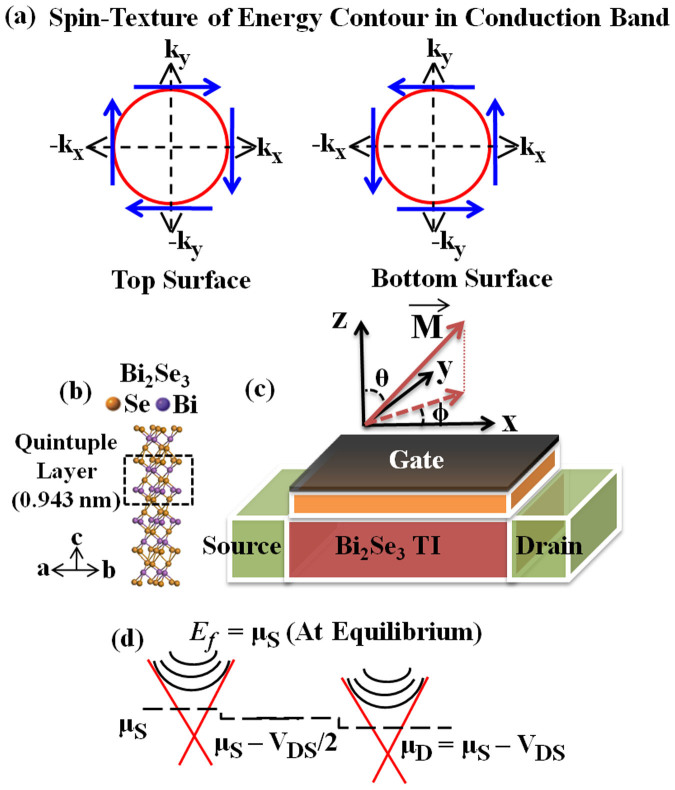
(a) Spin-texture in momentum space for conduction bands for top and bottom surface. Blue arrows are spin vectors, red circle is energy contour and black arrows denote momentum vectors. (b) Atomic structure of Bi_2_Se_3_ (3D-TI). 5 atomic layers constitute one quintuple layer (QL ~ 0.943 nm). (c) Device Structure for quantum transport modeling through Bi_2_Se_3_ slab. The axis are given over the device along with convention for polar representation of Magnetization vector (**M**) in this work. *θ* is polar angle and ∅ is azimuthal angle. Slab dimensions of 10 QL thickness – periodic width (uncoupled mode space) −65 nm channel length −10 nm contact on both side of channel, temperature of 0 K, a potential drop of 35 mV across channel (V_DS_) and Fermi-level (*E_f_*) = 0.075 eV has been considered as a representative case throughout this work unless specified otherwise. (d) Illustration of the potential distribution through the energy bands along the transport direction. *μ_S_* (*μ_D_*) is electrochemical potential at source (drain) end.

Bi_2_Se_3_^27^ is chosen as a representative 3D-TI in this study owing to its large bulk-bandgap of ~0.3 eV, which is largest among extant 3D-TIs, and hence, provides large energy window for transport through topological surface bands. The parameters for device Hamiltonian in eq. (1) are chosen to fit the experimental dispersions, and its validation is elaborated in our previous work^20^,^28^. Each infinite quintuple layer (x-y plane; see Fig. 1(b)) of Bi_2_Se_3_ is described in *p_z_* spin-orbital basis by *k*.*p* model by H_p_
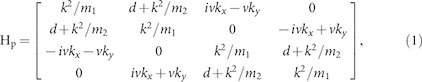
where *v* = 2.5 eV Å is the Fermi velocity, *m_1_* = 0.125 eV^−1^Å^−2^ and *m_2_* = −0.04 eV^−1^Å^−2^ are the orbital masses and the parameter *d* = −0.22 eV is introduced to generate a gap. In-plane wavevector *k* is computed as *k*^2^ = *k_x_*^2^ + *k_y_*^2^ where *k_x_* and *k_y_* are wavevectors along x-axis and y-axis respectively. The hopping between adjacent layers is described by tight-binding parameter *t_z_* = 0.35 eV in hopping matrix *T* as per eq. (2).
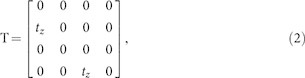
For modeling the transport in the *x*-direction (see Fig. 1(c)), *k_x_* wave-vector in eq. (1) is discretized via finite difference method (FDM) by substituting *k_x_* by *−i* *∂/∂x*. Transverse direction (*y*-axis) is modeled in uncoupled mode space (*k*-space (eigenmodes))for an infinitely wide channel (periodic condition) with no potential variation along the transverse direction^29^,^30^,^31^, to compute with reasonable computational resources^32^. Recursive-Green function (RGF)^33^ and Non-Equilibrium Green function (NEGF) algorithm are employed to describe the transport through a defect-free 3D-TI slab. To model the effect of infinitely or semi-infinitely long channel, computationally one or both of the terminal contacts are treated as if made of the same material as the channel^34^,^35^ This is done by applying open boundary condition at the contact for reflection less propagation of the plane wave and the contact self-energy is computed from the self-consistent solution of the surface green function^36^. This type of contact is referred as exTI (extended TI or extended channel) to distinguish it from other contact configurations. From the experiment's point of view, this refers to the scenario where terminals are far away from the surface probes to affect the measurements i.e. when voltage probes (VPs) (measurement probes) are sufficiently far from the current-injection probes (CPs) for very long channel device (typically in *μm* range). The measurement characterizes only the region between VPs, and captures the transport only in the TI material. Semi-infinite 3D-TI contacts are more appropriate to model such a system. On the other hand, modeling metallic contacts induces the effect of confinement and a hard wall boundary condition. When metallic (normal or ferromagnetic) contacts are applied at both ends, the system is referred to be a short-channel device. For such systems, the confinement and the type of contact is expected to influence the readings. Acoustic phonons scattering^20^ is modeled as self-energy in the channel and converged self-consistently. The key equations are summarized as follows:
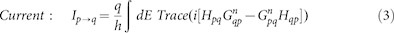

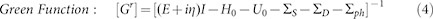

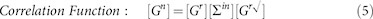

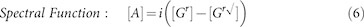

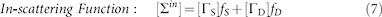

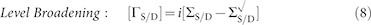

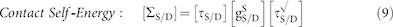

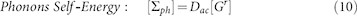

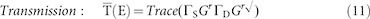
where ‘*p*’ and ‘*q*’ are two successive *y-z* surfaces between which the current is evaluated and *H_pq_* is hopping matrix from *p* to *q* planes. *H_0_* is the device Hamiltonian and *U_0_* is potential distribution in device. *D*_ac_ is the deformation potential for modeling acoustic phonons. τ_S/D_ is the coupling between the channel and the contacts, and *g^S^_S/D_* is the surface Green's function of the contact (source/drain). For the semi-infinite metallic contacts, *g^S^_S/D_* is phenomenologically calculated^37^ assuming constant density of states (*DOS_M_*^38^) (~0.008 eV^−1^) of metal and a contact-coupling coefficient (α), in range of 0 to 1, that describes the quality of contact with channel. The contact self-energy for metallic contacts therefore becomes
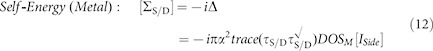
where, *I_Side_* is identity matrix of size, number of layer × basis size, between metallic contact and TI channel. For accurate modeling, an ab-initio computation is required to determine the hopping energy between Bi_2_Se_3_ and specific contact material. Nonetheless, as we show in the discussion section, the conclusions are is not sensitive to the exact value.

For ferromagnetic metal contact, *Δ* was magnetized by modifying the coupling for spin-up (down) by a factor of *1* + *(−)C_M_/100*, where *C_M_* is contact magnetization (in percent). Then depending on the magnetization vector, the unitary transformation^39^ was applied on *Δ*. Here, we would emphasize that coupling parameters for metallic contacts describe the transport only phenomenologically^37^ to help us to understand the underlying physics and neglects more complex effects like exchange interaction at the interface. The charge correction from Poisson equation has also been neglected because of the low-field condition^20^ (near-equilibrium transport).

## Results and Discussion

**Figure 2:**
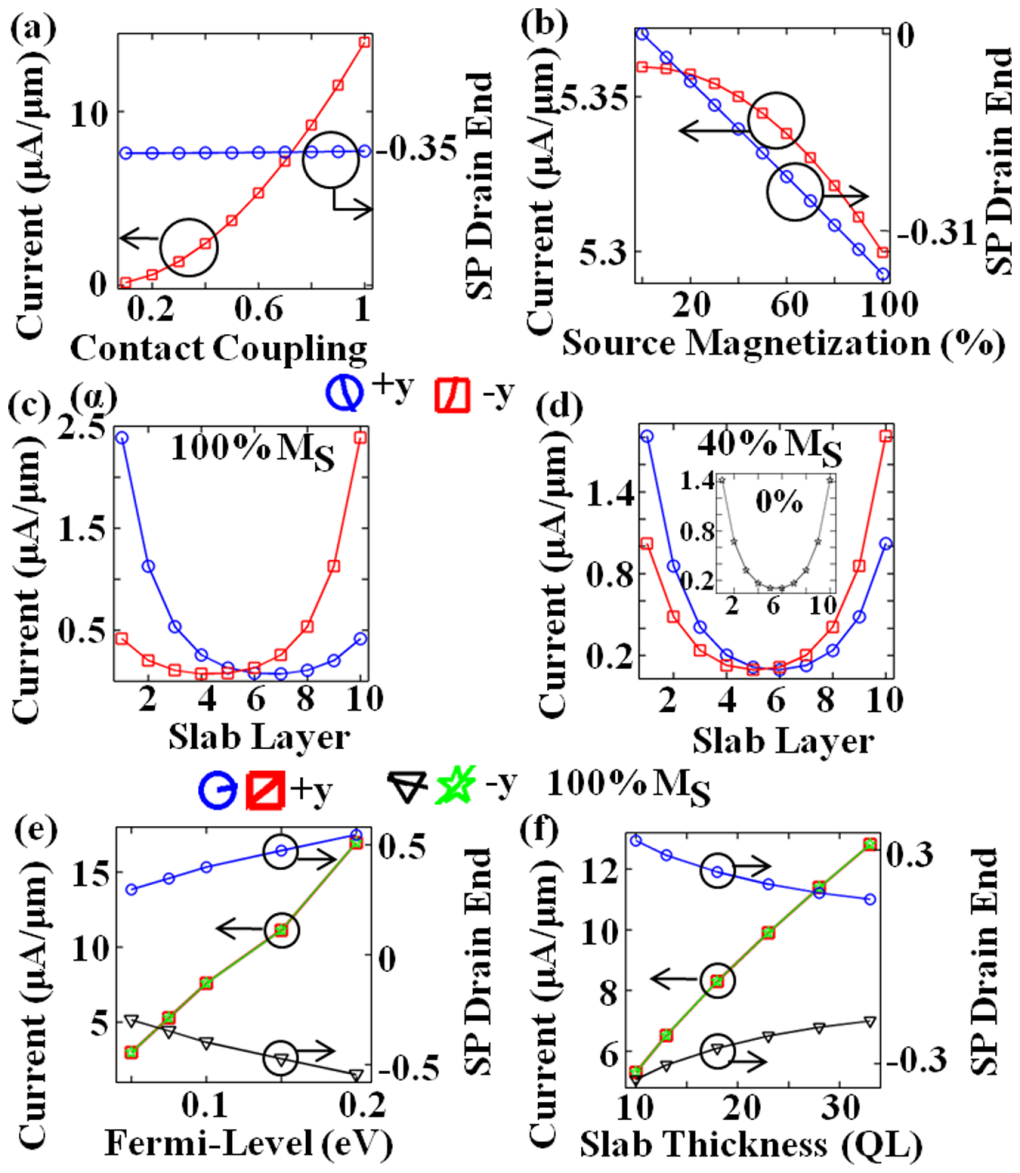
FM-TI-exTI system. Spin-Polarization (SP) at Drain end and current through slab over a range of (a) contact-coupling strength (α) for 100% ‘−*y*’ source magnetization (*M_S_*); (b) ‘−*y*’ *M_S_* for *α* = 0.6. From here on only *α* = 0.6 is considered for moderate quality of contact. Current distribution across slab layers for (c) 100% and (d) 40% *M_S_* along ±*y* axis. Note that current chiefly flows through layer with conducive spin-texture (see Fig. 1(a)). Inset shows the symmetric current distribution for 0% *M_S_*. SP at drain end and current for 100% *M_S_* along ±*y* axis over a range of (e) Fermi-level (*E_f_*) (f) slab thickness.

Firstly, we investigate a FM-TI-exTI system. Figure 2(a) shows the effect of quality of FM source contact-coupling (*α*) with the channel on the drain current and its spin-polarization (SP). ‘*α*’ is a material dependent parameter as each material (even NM) will have different coupling strength with the TI. With the improvement in the quality of the contact-coupling, metallic reservoirs can more easily inject electrons into the channel. This enhances the current through all layers. On the basis of Fig. 2(a), it is stressed that absolute value of *α* does not affect the conclusions qualitatively because the results only get scaled with the actual value of current. Therefore, for the subsequent discussion, we select the value of 0.6 for *α* i.e. a moderate quality contact. It is also observed that despite of electron injection from 100% (−*y*) magnetized source (*M_S_*), drain current is only ~34.7% spin-polarized because of non-zero current through sub-surface layers. We would also like to note that this phenomenon cannot be captured in the usual 2 × 2 Dirac-model used for modeling topological surface which normally overestimates this effect^40^. Since, only the normal mode (*k_y_* = 0) for electron energy very close to Dirac-point has complete *−y* spin orientation (see Fig. 1(a)), for higher energies the spin-vector starts to go out-of-plane^41^ and for larger transverse modes (*k_y_* ≠ 0) spin-vector has ±*x*-spin component, together they result in low SP. Furthermore, SP is independent of actual magnitude of current injection in this system because contact is uniformly coupled to all layers of the slab and hence, scales the current through each layer proportionally. A real contact, which probably can be simulated via ab-initio models, may slightly differ in this aspect because the contact coupling is material dependent and spin-injection efficiency into the channel depends on the contact-channel interface.

Next, Fig. 2(b) illustrates that SP scales directly with degree of source magnetization (*M_S_*), where 0% *M_S_* corresponds to Normal Metal. Therefore, a contact material with larger magnetization at source end should result in higher SP at the drain end. A small decrease in current is observed with increasing *M_S_*. It is due to mismatch between magnetized contact and TI channel, which increases with increasing *M_S_* because of different coupling with ±*y* spins for asymmetric injection. It is captured in the contact self-energy. Since, *M_S_* and *α* are material dependent parameter, although the qualitative effect is same, the observed magnitudes of drain current and SP will depend on the choice of FM (e.g. Fe or Co^9^).

Figure 2(c, d) next present a clear evidence of spin-momentum locking on the topological surface. For −*y* spin injection, current flows chiefly on the top-surface and on bottom-surface for +*y* electron spin in conformance with spin-texture (see Fig. 1(a)). In Fig. 2(c) (100% *M_S_*), note that there is a finite current through other layers, even on opposite surface. This can chiefly be attributed to two reasons. Firstly, the current in the channel is not 100% spin-polarized and transport has modes other than *k_y_* = 0. Secondly, topological behavior of the system forces the current to flow on the surfaces. Only very near to the source contact current is verified to be least on the opposite surface. Therefore, there is a small transition region after source-contact where current redistributes across layers, as elaborated later in the discussion, with conservation in *y-z* plane. Accordingly, going from full (100%) magnetization to weaker source magnetization of 40% in Fig. 2(d) the current becomes less anti-symmetric about middle layers. Subsequently, the effect of Fermi-level is shown in Fig. 2(e). For ballistic operation at 0 K in surface metallic bands of TI, the current through the device increases with increase in Fermi-level (*E_f_*) and channel-bias (*V_DS_*). For the operation in surface bands the increase in surface current is much more compared to sub-surface layers for both *E_f_* and *V_DS_*, unlike the effect of scaling contact-coupling *α*. Higher current through surface results in higher SP at the drain end (see Fig. 5(a) of Ref. 9) but of opposite polarity for ±*y* spin injection. Note that if the *E_f_* is eventually moved close to or in the bulk bands, then inspite of higher current, SP may reduce because of unpolarized bulk bands. Next, as illustrated in Fig. 2(f), the increase in slab thickness nevertheless reduces the fraction of current flowing through surface layer inspite of increase in total current due to current flow through more layers. This results in decreasing spin-polarization at the drain end with increasing slab thickness (see Fig. 5(b) of Ref. 9).

**Figure 3:**
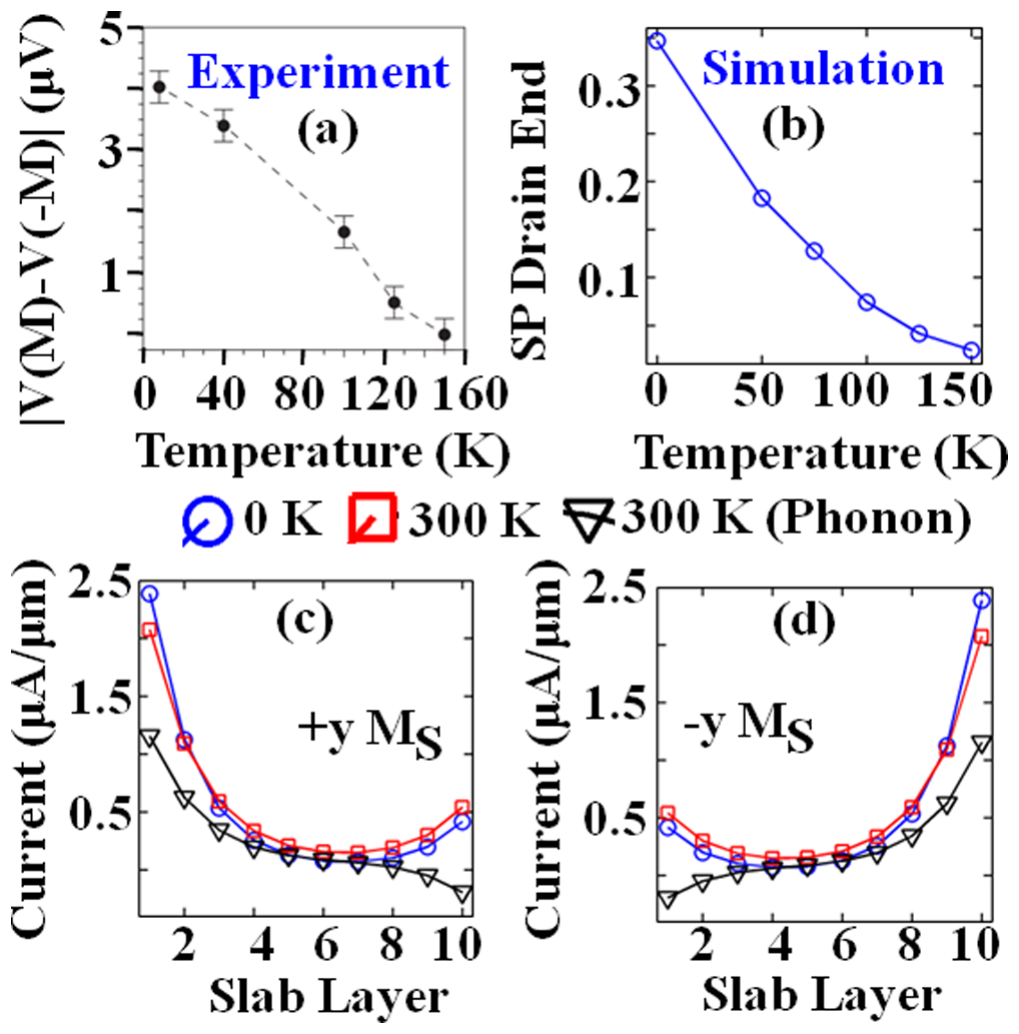
FM-TI-exTI system. (a) Experimentally observed trend for signature of spin-momentum locking on Bi_2_Se_3_, taken from Fig. 5(c) of Ref. 9 (Adapted by permission from Macmillan Publishers Ltd: [Nature Nanotechnology] (9), copyright (2014)). (b) Computed spin-polarization at drain end across temperature (without phonons) for +*y* (100%) *M_S_* contact. −*y* Magnetization produces same values but with opposite polarity. (c, d) Current distribution across slab layers for ballistic transport at 0 K (|*SP_Drain_*| ~ 34.7%) and 300 K (|*SP_Drain_*| ~ 0.43%) and with acoustic scattering at 300 K (|*SP_Drain_*| ~ 0.32%) for 100% source magnetization (*M_S_*) along (a) ‘+*y*’ and (b) ‘−*y*’ axis.

The analysis of effect of temperature reproduces the experimental data in Fig. 3(a, b) which showed that the signature of spin-momentum locking rapidly erodes with increasing temperature. Evaluation at room-temperature is thereafter illustrated in Fig. 3(c, d) for both ±*y* spin injection. Although the ballistic current through surface layer increases from 0 K to 300 K, the spread in Fermi-distribution drives the electron energies into higher energy states which are unpolarized^20^. This causes the degradation of the SP at drain end from 34.7% to mere 0.43%. Both current and SP further degrade when electron-phonon interactions are further taken into account. Besides phonon scattering, note that at higher temperatures the thermal effect on the magnetization of the ferromagnetic leads in an experiment may also result in the suppression of the spin polarization. However, since the ferromagnetic leads are quite large (60–80 μm) there will be minimal superparamagnetic effect. Hence, significant thermal effect on its magnetization would occur only close to its Curie temperature, which at above 1000 K for iron or cobalt is much higher than the temperature considered in our simulation. It would thus not be critical to the main conclusions drawn in this work, which focuses more on near zero Kelvin operation. Consequently, it is important to execute such experiments at low temperature (see Fig. 5 (c, d) of Ref. 9 where measurement signal was lost by 125 K) in order to suppress the phonon scattering and get reasonably spin-polarized current.

We have construed the recent experimental results for the electrical transport that observed spin-polarization resulting from spin-momentum locking on the surfaces of 3D-TI. This, however, pertains to a long-channel device and as stated earlier there could be more exciting phenomena at smaller channel lengths where physics at or due to contacts becomes more significant. Before discussing our observations for such devices, we briefly digress to investigate the effect of contacts more closely to develop a better understanding of underlying mechanism. In the subsequent discussion, *f_S_* (*f_D_*) is Fermi-distribution at source (drain), *E* is the energy grid for electron injection for energies from *μ_D_* to *μ_S_* for positive channel bias V_DS_. At 0 K, *f_S_*(*E* = *μ_S_*) = 0.5 otherwise it is 1 whereas *f_D_*(*E* = *μ_D_*) = 0.5 otherwise it is 0 on the energy grid. Therefore, drain contact injects charge (in-scattering) into the channel (see eq. (7)) only for *E* = *μ_D_*_._

**Figure 4:**
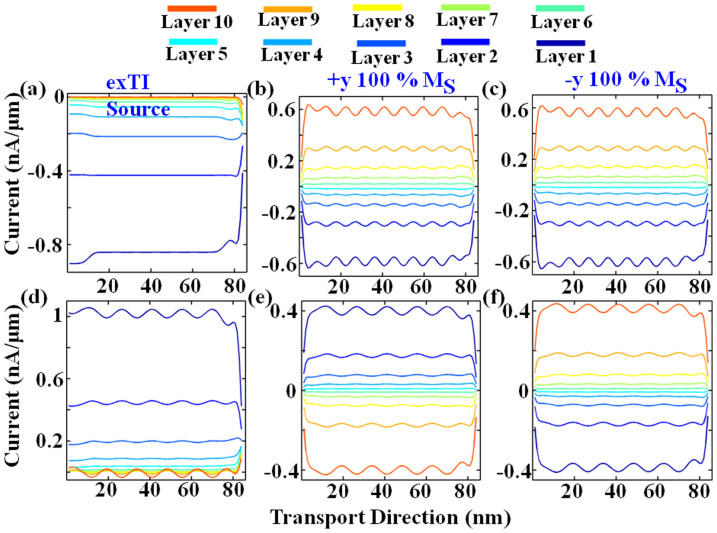
Current distribution along transport direction for (−*y*) 100% *M_D_* FM Drain for normal mode (*k_y_* = 0). Source contact-type is stated over each column. Operating condition is as follows: (a–c) *E* = *μ_S_*, *f_S_* = 0, *f_D_* = 1; (d–f) *E* = *μ_D_*, *f_S_* = 1, *f_D_* = 0. Refer text for symbols.

Consider the simplest case of only one energy point (see Fig. 4) for normal mode (*k_y_* = 0) electron transport for (*−y*) 100% M_D_ FM drain contact, because for normal mode spin-vector exactly aligns with in-plane contact magnetization, and three configurations of source contact i.e. exTI (extended-TI) and (±*y*) 100% *M_S_*. Firstly, to focus on the drain we set *E* = *μ_D_*, *f_S_*(*E*) = 0 (note that it is normally 1 for transport at 0 K), *f_D_*(*E*) = 1 in Fig. 4(a–c). It is observed that for exTI source, there is roughly no top layer current, whereas for ±*y* *M_S_* there is significant positive current through the top layer. For exTI configuration, there is a negative current (current direction opposite to voltage bias) in bottom layer in Fig. 4(a) because for injection from drain i.e. in *−k_x_* direction the bottom surface has the appropriate spin-momentum state (see Fig. 1(a)). A similar isolated injection from source (−*y* *M_S_*) results in entire current on top surface (not shown). For +*y* FM source, magnitude of top and bottom layer current is same but it shows the opposite polarity. For −*y* FM source, although magnitude of current simultaneously increases through both layers, there is more current through bottom layer. This indicates that magnetic contacts are causing some reflection in the current. For −*y* source, since source-contact has same phase as drain, the transmission through contact is higher and hence, there is weaker reflection compared to +*y* FM source configuration. Close examination reveals that even at the exTI source-contact there is a very small reflection current. Note that in Fig. 4(a–c) the injected spin from the drain is constant and only the reflection gets modified with source-contact type. Next, to focus on the source, we set *E* = *μ_S_*, *f_S_*(*E*) = 1, *f_D_*(E) = 0 (note that it is normally 0.5 for transport at 0 K) in Fig. 4(d–f) for injection from the source contact. Before examining the current distribution, we comment on two subtle observations that are evident from the plots: (a) the cyclic trend (oscillations) for surface layer current along transport direction due to the reflection from the drain contact; (b) the wavelength of oscillation is a function of electron energy (compare the two rows of sub-plots in Fig. 4) but nearly independent of channel length (verified by simulating from devices of various lengths from 30 nm to 120 nm), with lower energy electrons having longer wavelength as expected from De Broglie's relation. The amplitude of the oscillation is a function of degree of in-plane magnetization along y-axis and the percentage of magnetization (strength) (verified by simulating for entire range of polar and azimuthal angles). Now by appraising current distribution in Fig. 4(d–f), it is observed that for +*y* FM source positive current flows through the bottom layer (see from spin-texture in Fig. 1(a) that forward mode of bottom layer supports +*y* spin) and negative reflection current through the top layer. The magnitude of current is same although of opposite polarity. Here, we would note that this is equal only for *k_y_* = 0 mode (the simplest case under consideration). For −*y* FM source, positive current flows through the top layer and negative reflection current through the bottom layer. The magnitude of top current is greater than that of bottom (*measured via cursor in matlab, this may not be clearly visible from the figure*) again because of better phase matching at drain end which gives relatively weaker reflection current and higher transmission. Nevertheless, for exTI source, it is observed that there is positive current through bottom layer and close to zero current on top layer. Spin polarization shows that this current has +*y* spin. This is most counter-intuitive because it would be expected from spin-texture in Fig. 1(a) that −*y* drain spin-polarization matches with the forward moving state of top surface, therefore suggesting that current should flow through the top layer. Even a single energy level model with spin (2 × 2 matrix)^39^ with ‘βup’ and ‘βdown’ coupling for up and down spin respectively would imply that we have source coupling to both +*y* and *−y* spins (exTI source) whereas for the drain the coupling should only be finite for −*y* spin, and therefore −*y* spin should flow through top layer. Conversely, our calculation results indicate otherwise. This can be explained on the basis of transmission and reflection concept as follows. Consider that source injects ‘*I_1_*’ μA/μm of −*y* current on the top layer and ‘*I_0_*’ μA/μm of +*y* current on the bottom layer (in forward) direction. Because drain is −*y* magnetized, entire current on bottom layer is reflected through the top layer i.e. top layer has a reflection current of ‘−*I_0_*’ μA/μm. The current injected in the top layer, sees same phase (magnetization direction) in drain and has non-zero transmission (lower reflection). Therefore, ‘−(*I_1_* − *I_2_*)’ μA/μm current is reflected through the bottom layer. As a result, net current on the top layer is ‘*I_1_* − *I_0_*’ and on bottom it is ‘*I_0_* − *I_1_* *+* *I_2_*’. Since, TI contact injects roughly equal ‘*I_0_*’ and ‘*I_1_*’, we have nearly zero current on the top layer and ‘+*I_2_*’ μA/μm on the bottom layer. In general:
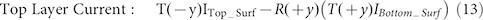

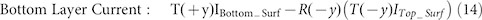
where, T (R) is transmission (reflection), in range of 0 to 1, of a particular spin from source to channel and *I_Top(Bottom)_Surf_* is surface current for TI system with exTI contacts (approximately without any reflection because some reflection will still be there caused by mode mismatch in non-equilibrium condition). Here, *T* and *R* are function of (i) strength of contact magnetization (for instance 40% or 100%), (ii) azimuthal angle, (iii) polar angles of each contact (i.e. type of magnetization), (iv) electron injection energy *E_i_*, and (v) *k_y_* mode. For (iv) and (v) the TI spin-texture and thus *I_Top(Bottom)_Surf_* depend on them. Also note that although eq. (13–14) only consider the top and bottom layer, the surface current can be affected by transmission and reflection for all sub-surface layers and therefore the expression is just for an empirical understanding of the underlying physics and lays the foundation for subsequent discussion. The exact modeling of eq. (13–14) is beyond the scope of current work.

**Figure 5:**
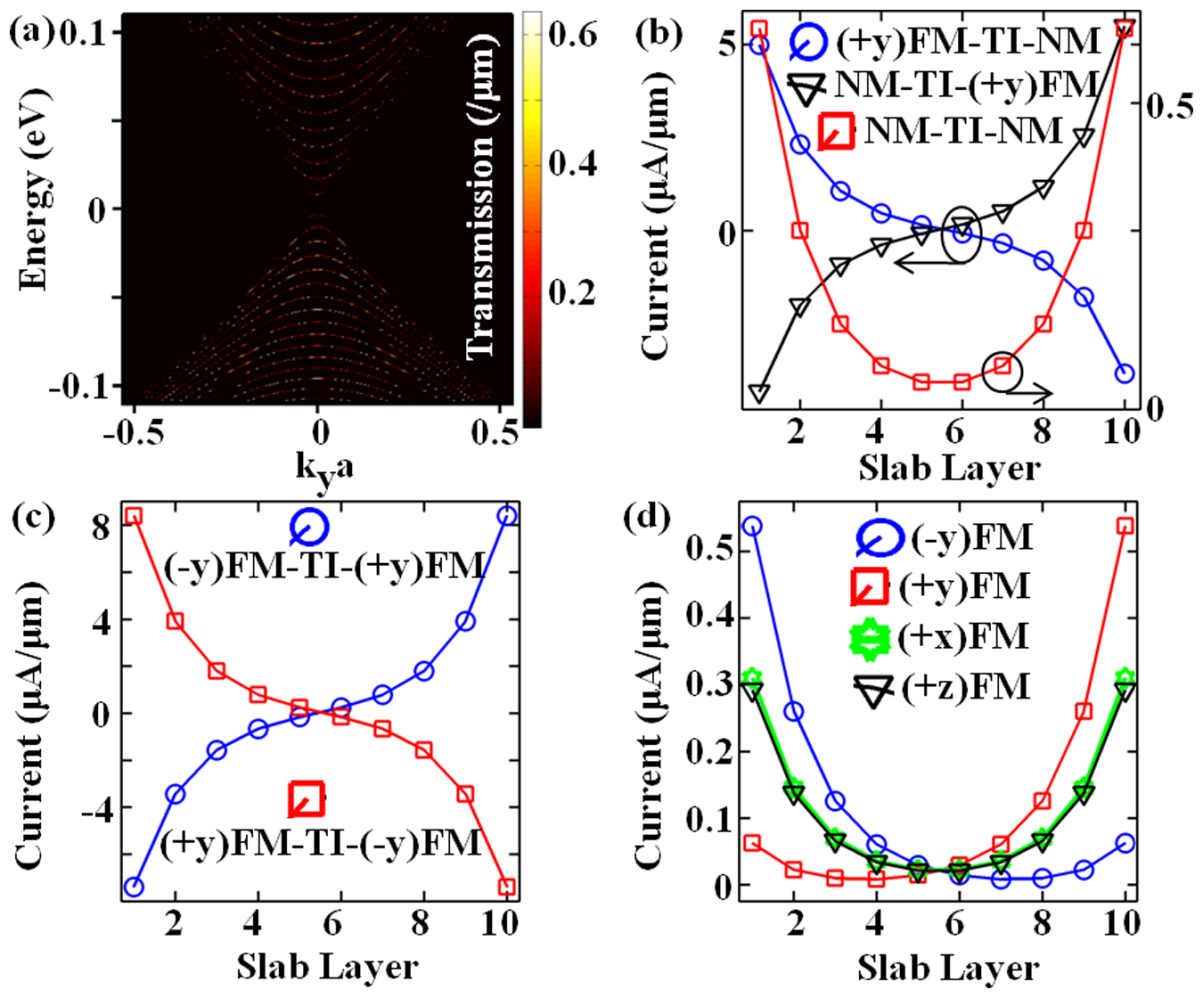
(a) Transmission distribution at equilibrium (*V_DS_* = 0 V) for normal metal contacts at both ends of the TI channel. Note that the confinement along the transport direction quantizes the bands which cannot be captured in energy-dispersion along transport direction (*x*-axis). Current distribution across slab layers for (b) three different combinations with normal metal contact, (c) with anti-phase ferromagnetic contacts, and (d) in-phase ferromagnetic contacts.

We now transition to short channel devices for which neither of the contacts can be modeled as exTI. Figure 5(a) illustrates transmission spectrum over finely discretized grid for energy and transverse modes (*k_y_*) for device in equilibrium condition. Unlike infinite channel length (contacts modeled as semi-infinite extended channel), the confinement along the transport direction (*x*-axis) quantizes the bands, and is therefore highly subjective to channel length, which cannot be captured in energy-dispersion along transport direction because energy-dispersion calculation presumes plane wave propagation along the corresponding wave-vector axis. This is, however, revealed clearly in transmission spectrum simulated over all relevant transverse and energy modes. The current depends on all five criterions discussed above for eq. (13–14) for both contacts. The transverse mode (*k_y_*) which contributes most for a given energy point on grid will depend on the choice of that E point. For high energies it will in principle be further from *k_y_* = 0 centre-point on *k_y_* grid, unless it is through sub-band edge at *k_y_* = 0, as illustrated from distribution of red-yellow spots in transmission spectrum. Furthermore, this can also be understood in terms of various cross-sectional energy contours from the conical frustum of energy-dispersion curve of surface conduction band and analyzing the permissible *k_x_* and *k_y_* modes in non-equilibrium. Note that at equilibrium it should be exactly at the intersection of chosen *k_y_* mode and energy-point on the sub-band illustrated in Fig. 5(a). Furthermore, as advanced later in discussion for Fig. 5(d), the distribution of dominant *k_y_* modes leads to some non-trivial counter-intuitive results because the overall result is governed by spin-momentum locking at these dominant modes instead of the normal mode (*k_y_* = 0) which was discussed above. Fig. 5(b) illustrates the effect of normal metal contact. If both contacts are normal-metal (red squares) indicating that we have symmetrical distribution of current about the middle layer, the most of which flows on surface layers as expected from topological properties of the 3D-TI. This distribution is same as that obtained by modeling both contacts as exTI, only the magnitude is different because of the difference in magnitude of contact coupling. For (+*y*) 100% FM source contact (blue circles) positive current flows through the bottom surface, in accordance with spin-texture, however, there is strong reflection current in top layer because backward moving state of top layer has +*y* spin-vector locked to −*k_x_* momentum on an average. Here we stress that net current along *y-z* plane is conserved throughout transport direction and total current is indeed positive. For NM source with (+*y*) 100% FM drain contacts (black triangles), the positive current flows through top layer and negative reflection through the bottom. This case is exactly opposite of the one discussed for Fig. 4(d) and so is the current distribution across layers. Fig. 5(c) then considers both contacts to be ferromagnetic but anti-phase with each other. Similarly, the current distribution is as expected from the spin-texture for each surface but with larger magnitudes because the forward moving state of one surface exactly matches with the backward moving state of opposite surface. The same phase of ferromagnetic contacts is considered in Fig. 5(d) (see supplementary for the effect of slab thickness on current distribution for anti-phase and in-phase contact configuration). ±*x* or ±*z* spin-injection can be equally resolved along ±*y* -axis and hence, we get the same current distribution as for NM or exTI source and drain contacts. Therefore, magnetization orientations along ±*x* or ±*z* for both or either contact is not expected to show any spin-momentum locking signature in transport experiments (see Fig. 3(e, f) of Ref. 9). For ±*y* in-phase magnetization, which should see small (high) reflection (transmission) at drain, we have the counter-intuitive results as pointed out earlier. If normal mode had dominated the transport, then as evident from Fig. 4(c, f), the largest fraction of positive current would have flown through the top layer and a negative current through the bottom surface. However, the spin-dependent transmission and reflection at *k_y_* modes away from *k_y_* = 0 point dominates the transport resulting in more current through top (bottom) surface for +*y* (−*y*) magnetization and a sizeable positive current through respective opposite surfaces.

**Figure 6:**
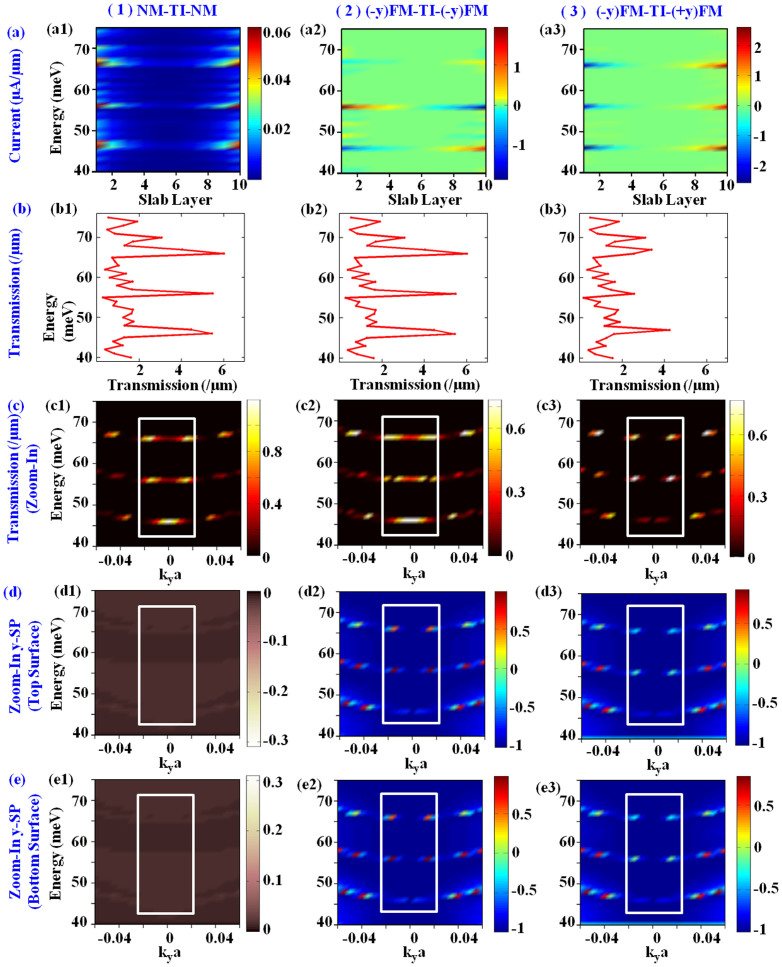
Energy resolved microscopic examination of current (a), Transmission (b), *k_y_*-resolved Transmission spectrum (c), spin-polarization of current through top (d) and bottom surface (e) layers for three sample cases (as stated over each column (1)–(3)) taken from Fig. 5. Zoom-in plots are illustrated instead of showing full-information to emphasize on the key features. Square box are drawn to draw attention to dominant modes (see text). Specifically, observe the confinement induced band quantization which limits the current through certain dominant energy and *k_y_* modes in (c). Also note that the current through surface layers is symmetrical about middle of the slab for NM contacts, but is spin-dependent for FM contacts. The net current integrated over all layers is always positive in above cases. Spin-polarization of certain dominant energy and *k_y_* modes in (d) and (e) decide the selection of topological surface for transport illustrated in (a). Note that NM contacts strictly comply with spin-texture diagram in Fig. 1. In contrast, for FM contacts the net solution atleast depends on magnetization axis of source and drain, sub-band quantization, transverse mode and injection energy.

To get deeper physical insight into this phenomenon by developing step-by-step understanding, Fig. 6 illustrates the energy resolved information for important quantum transport parameters for three sample contact configuration (NM contacts, FM contacts in in-phase and anti-phase 100% magnetization along *y*-axis) as follows: current (*I*) (Fig. 6(a)) as a function of slab layers (spatial distribution along *z*-axis), Transmission (*TE*) (Fig. 6(b, c)), and spin-polarization of the current (SP) for top (Fig. 6(d) and bottom surface (Fig. 6(e)) as a function of ky modes. Firstly, current distribution in Fig. 6(a) affirms the topological behaviour of the system by illustrating that the current is mainly flowing through top and bottom surface layers but through certain energy-eigenmodes. As illustrated by the red-yellow color in Fig. 6(a1), for NM contacts, the current flows equally through both surface layers, and sub-surface layers (light-blue color) have lower current contributions. Subsequently, Fig. 6(a2) and (a3) illustrate energy resolved current flow for FM contacts, where red and blue color, respectively, show strong positive and negative values. The respective energy integrated values result in trends observed in Fig. 5. Specifically, notice the energy dependent selection of layers in Fig. 6(a2) which corresponds to the exotic behavior observed in Fig. 5(d) for in-phase (−*y*) FM contacts. To demystify this observation, we now break down the transport parameters of eq. (3–11). Next, the transmission in Fig. 6(b) shows that the metallic source and drain contacts confine the channel to induce the discretization of bands and certain energy modes have higher contribution (see peaks) to the transport. We note that this band-quantization is not caused by the confinement along *z*-axis. Only z-axis termination (periodic width with semi-infinite contacts) results in Dirac-surface bands whose transmission and DOS are linear function of energy as shown in Refs. 20, 28. Figure 6(b) is further decomposed with respect to *k_y_* modes in Fig. 6(c). By comparing Fig. 6(c) with peaks of 6(b) we can determine the dominant *k_y_* modes (within square box) at dominant energy-modes. Fig. 6(c) also shows that the transmission is a function of contacts' (source and drain together) magnetization (compare color-bars and golden spots among three systems within the square box). This analysis which explains current distribution along energy-axis in Fig. 6(a) is insufficient to explain surface layer selection for different contact configuration. Therefore, Fig. 6(d, e) next examine the spin-polarization of current at each energy and *k_y_*-mode through top and bottom surface layer, with dominant modes enclosed in square box. For NM contacts in Fig. 6(d1) and (e1) observe that −*y* (+*y*) spins are flowing through top (bottom) surface as expected from spin-texture, but the values integrated over entire *y-z* plane would result in zero polarization for the net current. For −*y* FM source in Fig. 6(d2, d3, e2 and e3), the spin-polarization of current flowing through each surface is strongly influenced by drain's magnetization and can in fact be of opposite polarity for certain ky modes (positive (red) in Fig. 6(d2 and e2) while nearly zeros but negative (light bluish-green) for Fig. 6(d3 and e3)). In addition to FM contacts, this is probably due to combined effect of confinement induced band-quantization and the non-equilibrium condition which breaks the symmetry along x-direction. The *y*-spin component is opposite in adjacent quadrants in *k*-space for a given ky and therefore, spins for projections of Dirac bands on *k_y_*-axis in non-equilibrium, which may be larger for certain modes than others, may result in either of the polarities. Although a further study, for instance ab-initio modeling with FM contacts, might be needed to give the detailed understanding of this kind of phenomenon, our investigation in this work consequently reveals that the net solution for spin-polarization at least depends on magnetization axis of source and drain, sub-band quantization, ky and Ei, and hence strongly influences the Transmission (*T*) and Reflection (*R*) coefficients in eq. (13–14). Therefore, from microscopic dissemination of quantum information, we note that for ferromagnetic contacts the current distribution is highly subjective to the choice of Fermi-level, bias (determines electron injection-energy) and contact magnetization, which may result in exotic observations for current distribution across layers of 3D-TI. Next, we observe that negative top surface current can be driven for certain configurations of metallic contacts (see Fig. 5(b, c)) for short channel devices. Therefore, the experimental observation of negative resistance for surface transport on thin slabs of potential candidates for 3D-TI can serve as a simple way to verify spin-momentum locking and hence validate the existence of topological insulator.

Finally, we would like to note that presence of helical surface state is necessary but not the sufficient condition for detecting a TI because the count of Dirac cones must also be unraveled. More specifically, Z2 class^2^ of TI has odd number of Dirac cones whereas another class called topological crystalline insulator (TCI)^42^ has even number^43^,^44^ of Dirac cones with same helicity for a given surface. Since only Bi_2_Se_3_ has been studied in this work, which belongs to Z2 class, we could be right in suggesting our method for the electrical detection of the topological insulators for this system only. Hence, as part of future work we would suggest to further mature this methodology for conclusively predicting topological insulators via electrical detection.

## Conclusion

In summary, we address the issue of influence of different contact types and configurations on current flow through thin Bi_2_Se_3_ 3D-TI. Specifically, we examined the current distribution across layers as it flows through the device for the extended-channel, normal metal and ferromagnetic metal contacts. Our model is shown to explain the recent experimental work^9^ on electrical detection of spin-momentum locking on topological surface. For experimental observations, it is suggested to perform the experiment on thin samples at low temperatures with magnetic contacts of high-polarization. We also show that for short channel devices the spin-dependent transmission and reflection at the contacts can result in observations seemingly counter-intuitive from simple spin-texture understanding of the topological surfaces. For the anti-phase magnetization between terminal contacts, along the y-axis, negative surface current can be generated which may provide a simple mechanism to validate topological insulators via quantum transport experiments by observing negative surface resistance. Recently various optical^45^,^46^, electrical^47^ and magnetic^48^ techniques have been established for selectively probing edge and surface transport of materials and may help in validating the predictions made in this work. Furthermore, for the in-phase configuration, it is shown that confinement and hence induced quantization of energy and momentum modes may result in current distribution antagonistic to trend expected from general understanding of spin-texture. The comprehensive understanding of transport in 3D-TI with ferromagnetic contacts should expedite the development of novel spintronic devices based on topological insulators.

## Author Contributions

G.G. performed the computations and wrote the manuscript. G.G., M.B.A.J. and G.L. critically analyzed the data. All authors reviewed the manuscript. G.L. also managed the overall execution of the project.

## Supplementary Material

Supplementary Information Supplementary information
