# Supplementary material for: Contact Effects in thin 3D-Topological Insulators: How does the current flow?
Source: Sci Rep. 2015 Mar 30;5:9479. doi: 10.1038/srep09479 (PMC4377578; doi:10.1038/srep09479)
Supplement: Supplementary Information [file srep09479-s1.doc]

Supplementary Material for: Contact Effects in thin 3D-Topological Insulators: How does the current flow?

Gaurav Gupta, Mansoor Bin Abdul Jalil, and Gengchiau Liang

*Department of Electrical and Computer Engineering, National University of Singapore, Singapore 117576*

This supplementary document provides a supporting plot for layer dependence of short-channel device with anti-phase and in-phase ferromagnetic contacts to emphasize the effect of contact magnetization and confinement along the transport direction. The microscopic dissemination of quantum information resolved in energy (*E*) and transverse-mode (*k_y_*) space reiterates that the current distribution for short-channel device with FM contacts is highly subject to the electronic and physical operating condition which may result in exotic observations for current distribution across layers of 3D-TI.

Figure S1(a) shows that for thickness ranging from 10QL-28QL, the current distribution across quintuple layers of the Bi_2_Se_3_ topological insulator with anti-phase ferromagnetic contacts show the same qualitative trend as Fig. 5(c) of main text. The positive current is flowing on the bottom layer while the negative current is through top surface layer, which is in accordance with spin-texture for top and bottom surface shown in Fig. 1(a) of main text. The transmission distribution resolved in E and *k_y_*-space in Fig. S1(b-f) for different slab thickness illustrates increase in the transmission modes in the operating energy window for the transport. These plots furthermore illustrate the quantization of momentum-modes due to longitudinal confinement and clearly show that normal mode (*k_y_* = 0) may or may not be the dominating one (also see discussion for Fig. 6 in main text). The absolute quantity of current flowing through each layer therefore depends on these quantized energy levels, the ky mode and its distribution across layers. However, for Fig. S1(g) for in-phase ferromagnetic contacts it is observed that it is possible to have an exotic current distribution, akin Fig. 5(d) of main text, for certain thickness. This again depends on the dominating *k_y_* modes as illustrated via Fig. S1(h-l), which may be different from the set obtained for anti-phase contact configuration (compare transmission plots in two columns), and their spin-polarization (see Fig. 6 and corresponding discussion in main text). We however acknowledge that due to extremely sensitive operating conditions like Fermi-level, bias, channel length, slab thickness and temperature, which governs the quantization of both energy and momentum modes and their spin-polarization, it may be a challenging experiment.


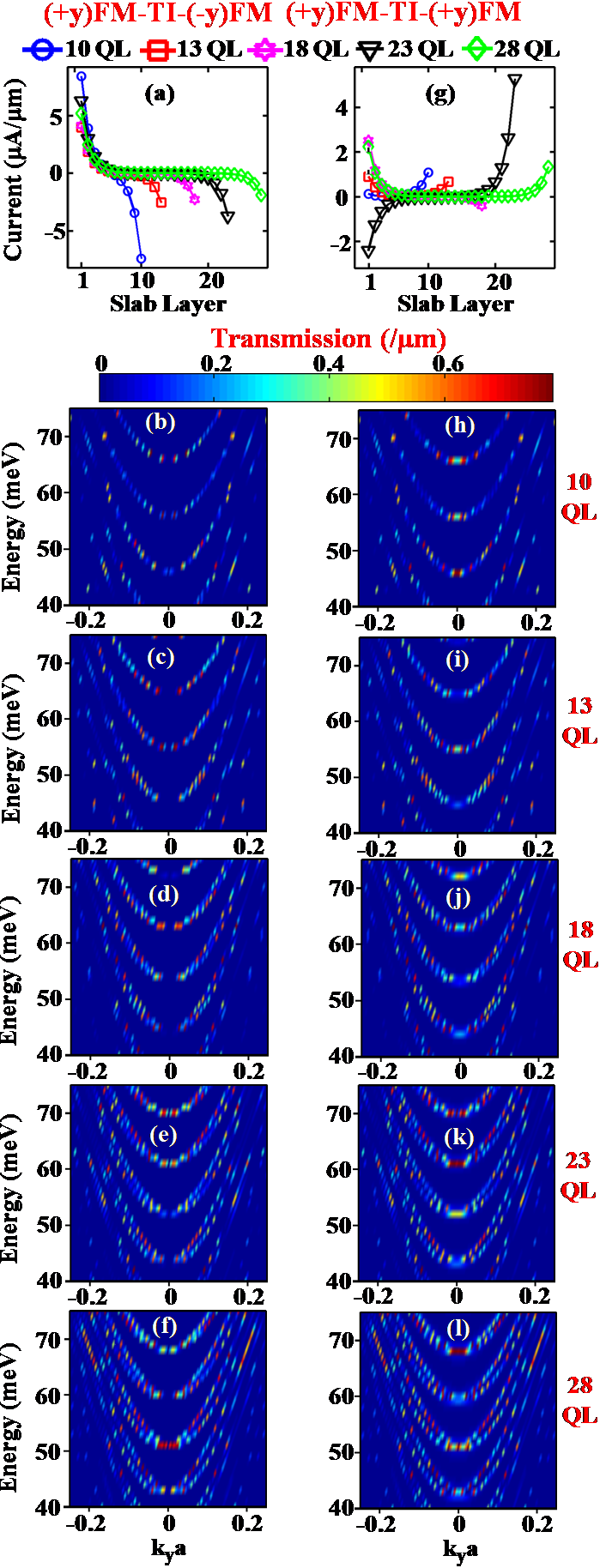


**Figure S1**. Transport at 0 K for *V_DS_* = 35 mV through 65 nm long TI channel with 100 % polarized ferromagnetic contacts at both ends. Source terminal is magnetized along ‘+*y*’ whereas drain terminal is magnetized along ‘−*y*’ (a-f) and ‘+*y*’ (b-l). (a, g) Current distribution across slab layers of different thicknesses. (b-f, h-l) Energy and transverse mode *k_y_a* resolved Transmission corresponding to current distribution in respective columns for thickness stated on right side in each row.
